# Suicide and Accidental Death Among Women With Primary Ovarian Cancer: A Population-Based Study

**DOI:** 10.3389/fmed.2022.833965

**Published:** 2022-03-16

**Authors:** Ying Chen, Kaixu Yu, Jiaqiang Xiong, Jinjin Zhang, Su Zhou, Jun Dai, Meng Wu, Shixuan Wang

**Affiliations:** ^1^Department of Obstetrics and Gynecology, Tongji Hospital, Tongji Medical College, Huazhong University of Science and Technology, Wuhan, China; ^2^Department of Orthopedics, Tongji Hospital, Tongji Medical College, Huazhong University of Science and Technology, Wuhan, China; ^3^Department of Obstetrics and Gynecology, Zhongnan Hospital of Wuhan University, Wuhan, China

**Keywords:** ovarian cancer, suicide and accidental death, histological subtypes, pelvic exenteration, risk factors

## Abstract

**Background:**

Women with ovarian cancer had the highest suicidal rate among all patients with gynecological malignancies, but no large studies about suicide and accidental death for women with ovarian cancers in detail were conducted. We aimed to determine the relative risk of suicide and accidental death among patients with ovarian cancer to that of the general population, and to identify risk factors associated with suicide and accidental death.

**Methods:**

Data are from the SEER (surveillance, epidemiology, and end results) cancer registry of women diagnosed with ovarian cancer data from 18 registries for the years 1973–2016. The study population comprised 149,204 patients after inclusion and exclusion criteria were applied. Standardized mortality ratios (SMRs) were calculated and Fine-Gray models were fitted to identify risk factors associated with suicidal and accidental death among cancer patients, with stratifications on demographic and tumor-related characteristics.

**Results:**

Women with ovarian cancer had a higher risk of suicide and accidental death than the cancer-free group [SMR = 1.86; 95% CI (1.54–2.25) and SMR = 1.54; 95% CI (1.39–1.71)]. Subgroup analysis indicated that only patients with type II epithelial ovarian cancer [SMR = 2.31; 95% CI (1.83–2.91)] had an increased risk of suicide, and those with type I and type II epithelial ovarian cancer [SMR = 1.65; 95% CI (1.39–1.97) and SMR = 1.49; 95% CI (1.30–1.70)] were at a higher risk of accidental death. Patients with ovarian cancer who were younger, white, diagnosed with high-grade, non-metastatic cancer and pelvic exenteration were at a higher risk of suicide. The advanced age, earlier year of diagnosis, and non-metastatic cancer were associated with a higher risk of accidental death. Additionally, pelvic exenteration increased the risk of suicide but not the risk of accidental death among women with primary ovarian cancer.

**Conclusions:**

Women with ovarian cancer had a higher risk of suicide and accidental death compared with the general population. The findings suggested that clinicians should identify high-risk subgroups of ovarian cancer patients for suicide and accidental death as early as possible, with appropriate prevention strategies.

## Introduction

Suicide is the act of intentional death, which is the tenth leading cause of death in the US, claiming the lives of more than 48,000 individuals in 2018 (1). It not only represents an individual tragedy but also takes a staggering toll on global public health. Accidental death is an unnatural death that is caused by unintentional injury. The hitherto unknown suicidal deaths may be misclassified as death from accidental or unintentional injury (2, 3). Both suicide and accidental death are regarded as external causes of death, and they share common risk factors such as physical, social, and emotional dysfunction (4). Though the 5-year survival rates of cancer patients have increased over the past decades, the risk of dying from suicide and accidental death for those patients remains elevated over time (5). Epidemiological studies have shown that the rate of death from suicide and unintentional injury in cancer patients was 1.9 (5) and 1.6 (6) times those of the general population, respectively.

Gynecologic cancers represent a major problem affecting women's health. Kristy et al. reported that women with gynecological malignancies were at a higher risk of suicide compared with those diagnosed with non-gynecological malignancies (7). Among gynecologic cancers, the patients with ovarian cancer had the highest suicidal rate among patients with gynecological malignancies (8). It was estimated that approximately 75% of ovarian cancer occurs as an advanced disease (9) because of the lack of effective screening methods and non-specific symptoms (10). This resulted in a high recurrence and mortality rate in ovarian cancer patients. Severe pain, high recurrence rate, intense treatment, and inferior quality of life from the disease itself and adverse effects of treatment often caused serious depression and anxiety among patients with ovarian cancer, which could put them at high risk for suicide and accidental death (11).

Previous studies have shown higher suicide rates in ovarian cancer patients compared with other gynecological malignancies (8), however, a direct comparison of the risk of suicide and accident injury especially among ovarian cancer with the general population has not been undertaken, and there is currently no literature on characteristics, incidence, and risk factors for suicide and accidental death in women with ovarian cancers in details.

As such, to address the current lack of evidence, we conducted a population-based analysis of the suicide and accidental death among patients with ovarian cancer. The objective of this study was to determine the relative risk of suicide and accidental deaths among ovarian cancer patients compared with the general population and to identify demographic and tumor-related characteristics, such as age at diagnosis and histological subtypes, that are associated with a particularly high risk of suicide and accidental deaths among ovarian cancer patients.

## Materials and Methods

### Patient Selection

The Surveillance, Epidemiology, and End Results (SEER) database (12) was established by the National Cancer Institute, covering ~28% of the US population. The Public Use version of data collected from the SEER18 registries (12) between January 1, 1973, and December 31, 2016, was used for this study. As a comparison, the mortality data of the general US population collected by the National Center for Health Statistics spanning from 1969 to 2016 was used.

### Study Population and Study Variables

**Figure 1:**
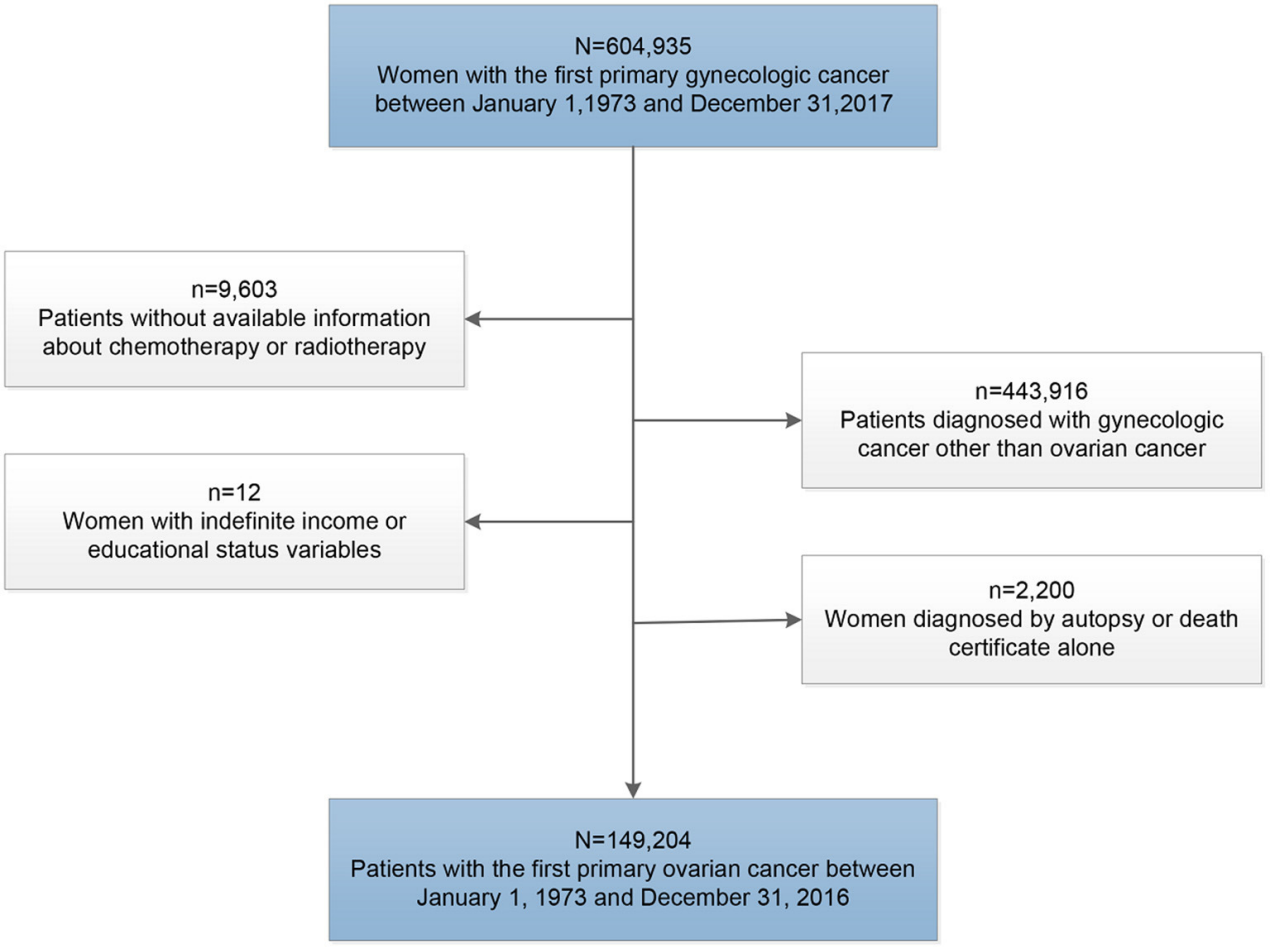
Flow diagram of patient selection within the SEER 18 registries between 1973 and 2016.

To avoid the influence of the second primary tumors on the outcome of cancer patients, we only included patients diagnosed with the first primary ovarian cancer at the beginning of this study. Patients were excluded if the diagnosis was made at autopsy or obtained solely from the death certificate, and those without definite data on age at diagnosis, income, educational level, and survival time were also excluded in this study (Figure 1).

Available data about demographic characteristics from the SEER database included age at diagnosis, race, and calendar year of diagnosis. The tumor-related variables encompassed pathological type, stage, and grade. Income (median family income) and educational level (percentage of residents >25 years of age with at least a bachelor's degree) were categorized into quartiles. Residence types (rural/urban) were obtained at the county level from the SEER program. Data on ovarian cancer treatment, follow-up time, and cause of death were also available. Ovarian cancer was categorized into five histological subgroups according to the International Classification of Disease for Oncology third revision (ICD-O-3) codes: type I epithelial, type II epithelial, germ cell, sex cord-stromal, and other (13, 14). Surgeries for ovarian cancer were categorized into “Oophorectomy without hysterectomy,” “Oophorectomy with hysterectomy,” “Surgery, not otherwise specified (NOS),” “None,” “Debulking,” and “Pelvic Exenteration.”

Patients were considered to have committed suicide if the cause of death variable was coded as “Suicide and Self-inflicted Injury (50220)” including International Classification of Diseases, Eighth Revision codes (ICD-8): 950-959, ICD-9: 950-959, and ICD-10 codes U03, X60-X84, and Y87.0. Patients were considered to have committed accidental death if the cause of death variable was coded as “Accidents and Adverse Effects (50210)” including ICD-8: 800-949; ICD-9: 800-949; and ICD-10: V01-X59 and Y85-Y86.

### Statistical Analysis

The number of suicides or accidental deaths divided by person-years of survival was calculated as the mortality of suicidal or accidental death. Among subgroups of cancer patients stratified by different characteristics, SMRs were calculated as the ratios of observed to the expected number of deaths (5, 6, 15), which provided the relative risk of death from suicide and accident injury for cancer patients compared with all general US population with the same distribution of age, sex, and race. The observed values represented the number of suicides or accidental deaths in patients with ovarian cancer, and the expected values represented the number of individuals who died by suicide or accident in the general population. A 5-year age range was used for standardization, and the 95% CI of SMR was determined by using the Poisson distribution approximation. Interaction tests were further carried out to investigate the potential differential effect of cancer subtypes on suicide risk during different follow-up periods. Given that other causes of death were considered as competing events, we also designed the Fine-Gray model to identify demographic and tumor-related characteristics associated with a higher risk of suicide and accidental death. Observations were censored if patients did not die from suicide or accidental death at the time of the last follow-up. The survival time recorded as 0 month in the SEER database was converted to one-half of a month based on accepted epidemiologic practices (15).

All statistical tests were two-sided, and values with *P* < 0.05 were considered statistically significant. The SEER database was accessed using SEER^*^Stat software 8.3.8 (12). The calculation of SMRs, cumulative incidence curves, Gray tests, and interaction tests were conducted in R version 3.51 statistical software. These histograms summarizing the results of subgroup analyses were drawn by Microsoft Excel and GraphPad Prism 8.0.

## Results

### Incidence of Suicide and Accidental Death Among Patients With Ovarian Cancer

**Figure 2:**
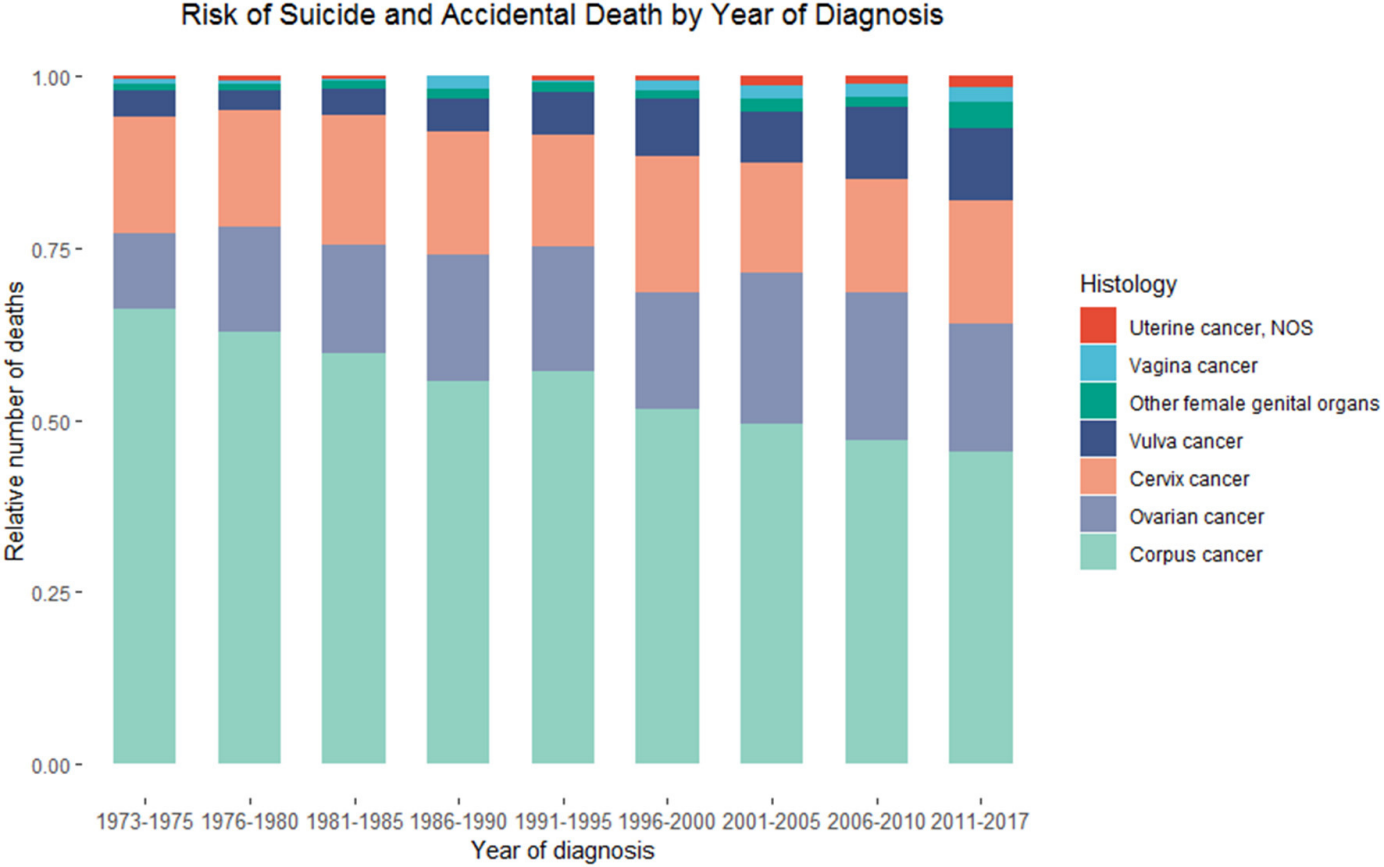
Death due to suicide and accidental injury among patients with primary malignant ovarian cancer in SEER database by calendar year of death.

A total of 107 suicidal and 373 accidental deaths occurred among 149,204 patients with ovarian cancer, followed by 768,840.17 person-years (Table 1). The suicide rate was 13.92/100,000 person-years and the accidental death rate was 48.51/100,000 person-years among ovarian cancer patients. Women with ovarian cancer had an 86% higher risk of suicidal death [SMR = 1.86; 95% CI (1.54–2.25)] and 54% higher risk of accidental death [SMR = 1.54; 95% CI (1.39–1.71)] than the general US population with the same distribution of age, sex, and race. The number of suicide and accidental deaths among patients with ovarian cancer ranks second in patients with gynecologic cancers, between uterine cancer and cervical cancer. From 1973 to 2017, the relative number of suicide and accidental deaths decreased gradually in patients with uterine cancer and unchanged among patients with cervical cancer, while the relative number of suicide and accidental deaths remained elevated among patients with ovarian cancer (Figure 2).

**Table 1:** The risk of suicide and accidental death among patients with ovarian cancer diagnosed between January 1, 1973 and December 31, 2016.

**Variable**	**No. of patients, ***n*** (%)**	**Person-years**	**Suicides^a^**			**Accidental deaths^b^**		
			**No. of deaths, ***n*** (%)**	**Mortality rate^c^**	**SMR (95%CI)^d^**	**No. of deaths, ***n*** (%)**	**Mortality rate^c^**	**SMR (95%CI)^d^**
**Age**								
0–50 years old	31,258 (21)	291,028.79	27 (25)	9.28	1.25 (0.86–1.83)	77	26.64	1.36 (1.09–1.70)
50+ years old	117,946 (79)	477,811.38	80 (75)	16.74	4.52 (3.63–5.63)	296	61.95	1.60 (1.43–1.79)
**Race**								
White	126,765 (85)	656,766.63	97 (91)	14.77	1.82 (1.50–2.23)	332 (89)	50.55	1.54 (1.38–1.71)
Black	11,231 (8)	47,666.21	2 (2)	4.20	1.68 (0.42–6.73)	26 (7)	54.55	1.87 (1.27–2.75)
American Indian/AK Native, Asian/Pacific Islander	10,762 (7)	61,631.83	8 (7)	12.98	2.74 (1.37–5.48)	14 (4)	22.72	1.23 (0.73–2.08)
Unknown	446 (0)	2,775.50	0 (0)	0.00	0.00	1 (0)	36.03	2.14 (0.30–15.21)
**Year**								
1973–1983	16,667 (11)	142,227.92	23 (21)	16.17	2.10 (1.40–3.17)	77 (21)	54.14	2.06 (1.64–2.57)
1984–1994	21,941 (15)	166,493.13	25 (23)	15.02	2.00 (1.35–2.96)	89 (24)	53.46	1.85 (1.51–2.28)
1995–2005	47,111 (32)	274,347.13	30 (28)	10.94	1.47 (1.02–2.10)	124 (33)	45.20	1.39 (1.17–1.66)
2006–2016	63,485 (43)	185,772.00	29 (27)	15.61	2.15 (1.49–3.09)	83 (22)	44.68	1.24 (1.00–1.53)
**Education^e^**								
High	46,980 (31)	264,741.88	42 (39)	15.86	2.12 (1.57–2.87)	119 (32)	44.95	1.46 (1.22–1.75)
Medium	52,495 (35)	269,128.08	35 (33)	13.00	1.75 (1.26–2.44)	135 (36)	50.16	1.62 (1.37–1.92)
Low	49,729 (33)	234,970.21	30 (28)	12.77	1.71 (1.19–2.44)	119 (32)	50.64	1.55 (1.29–1.85)
**Income^e^**								
High	49,603 (33)	276,519.21	43 (40)	15.55	2.05 (1.52–2.77)	125 (34)	45.20	1.46 (1.22–1.74)
Medium	45,261 (30)	238,441.83	30 (28)	12.58	1.66 (1.16–2.38)	116 (31)	48.65	1.56 (1.30–1.87)
Low	54,340 (36)	253,879.13	34 (32)	13.39	1.85 (1.32–2.59)	132 (35)	51.99	1.62 (1.36–1.92)
**Residence**								
Metropolitan	131,445 (88)	673,584.54	97 (91)	14.40	1.93 (1.58–2.35)	324 (87)	48.10	1.53 (1.37–1.70)
Non-metropolitan	16,093 (11)	79,892.33	6 (6)	7.51	0.97 (0.44–2.16)	44 (12)	55.07	1.64 (1.22–2.21)
Unknown	1,666 (1)	15,363.29	4 (4)	26.04	4.60 (1.73–12.25)	5 (1)	32.55	1.64 (0.68–3.94)
**Grade^f^**								
Low	29,892 (20)	248,906.46	18 (17)	7.23	0.94 (0.59–1.49)	104 (28)	41.78	1.48 (1.22–1.80)
High	59,048 (40)	251,698.00	45 (42)	17.88	2.37 (1.77–3.17)	111 (30)	44.10	1.32 (1.10–1.59)
Unknown	60,264 (40)	268,235.71	44 (41)	16.40	2.29 (1.70–3.08)	158 (42)	58.90	1.81 (1.55–2.11)
**Stage**								
Localized	30,442 (20)	325,990.54	32 (30)	9.82	1.31 (0.92–1.85)	134 (36)	41.11	1.50 (1.27–1.78)
Regional	17,588 (12)	105,362.54	16 (15)	15.19	2.02 (1.24–3.30)	47 (13)	44.61	1.40 (1.05–1.86)
Distant	91,669 (61)	305,955.33	49 (46)	16.02	2.15 (1.63–2.85)	168 (45)	54.91	1.59 (1.37–1.85)
Unknown	9,505 (6)	31,531.75	10 (9)	31.71	4.52 (2.43–8.39)	24 (6)	76.11	1.82 (1.22–2.72)
**Surgery**								
None	29,246 (20)	35,362.29	15 (14)	42.42	6.75 (4.07–11.20)	53 (14)	149.88	2.32 (1.77–3.04)
Debulking	36,959 (25)	140,717.75	20 (19)	14.21	1.89 (1.22–2.92)	55 (15)	39.09	1.15 (0.88–1.50)
Oophorectomy without hysterectomy	14,606 (10)	102,602.71	7 (7)	6.82	1.09 (0.52–2.28)	39 (10)	38.01	1.28 (0.93–1.75)
Oophorectomy with hysterectomy	41,072 (28)	293,544.42	33 (31)	11.24	1.42 (1.01–2.00)	118 (32)	40.20	1.37 (1.14–1.64)
Pelvic Exenteration	2,370 (2)	10,111.83	3 (3)	29.67	3.88 (1.25–12.03)	5 (1)	49.45	1.59 (0.66–3.81)
Surgery, NOS	17,833 (12)	173,819.00	22 (21)	12.66	1.66 (1.09–2.52)	85 (23)	48.90	1.85 (1.50–2.29)
Unknown	7,118 (5)	12,682.17	7 (7)	55.20	7.85 (3.74–16.46)	18 (5)	141.93	3.41 (2.15–5.41)
**Chemotherapy**								
None/unknown	58,447 (39)	339,837.00	52 (49)	15.30	2.07 (1.58–2.72)	190 (51)	55.91	1.73 (1.50–1.99)
Yes	90,757 (61)	429,003.17	55 (51)	12.82	1.70 (1.31–2.22)	183 (49)	42.66	1.39 (1.20–1.60)
**Radiation**								
None/unknown	143,841 (96)	723,213.58	99 (93)	13.69	1.84 (1.51–2.24)	354 (95)	48.95	1.54 (1.39–1.71)
Yes	5,363 (4)	45,626.58	8 (7)	17.53	2.20 (1.10–4.40)	19 (5)	41.64	1.65 (1.05–2.58)
**All**	149,204	768,840.17	107 (100)	13.92	1.86 (1.54–2.25)	373 (100)	48.51	1.54 (1.39–1.71)

*SMR, standardized mortality ratio; CI, confidence intervals*.

a *International Classification of Diseases, Eighth Revision codes (ICD-8): 950-959; International Classification of Diseases, Ninth Revision codes (ICD-9): 950-959; and International Statistical Classification of Diseases and Related Health Problems, Tenth Revision (ICD-10) codes U03, X60-X84, and Y87.0 and recode 50220*.

b *ICD-8: 800-949; ICD-9: 800-949; and ICD-10: V01-X59 and Y85-Y86 and recode 50210*.

c *Per 100,000 person-years*.

d *In brief, SMRs were estimated as the ratios of observed to expected number of deaths. The observed number of deaths represents the total number of deaths from suicide or accidental injury among patients with primary ovarian cancer. To obtain the expected number of deaths, we derived the stratum-specific mortality rates of suicide or accidental injury of the general US population and calculated the person-years of relevant strata in the cancer group. The stratum-specific expected number of deaths was estimated as the product of mortality rate in the cancer-free control and the person-years in the cancer group. The total expected number of deaths was a summation of all the expected number of deaths across the strata*.

e *Income (median family income) and educational level (percentage of residents >25 years of age with at least a bachelor's degree) were categorized into quartiles*.

f *The variable of low grade included “Well differentiated; Grade I” and “Moderately differentiated; Grade II,” and the variable of high grade included “Poorly differentiated; Grade III” and “Undifferentiated; anaplastic; Grade IV”*.

### Demographic Characteristics Associated With Increased Incidence of Suicide and Accidental Death

**Figure 3:**
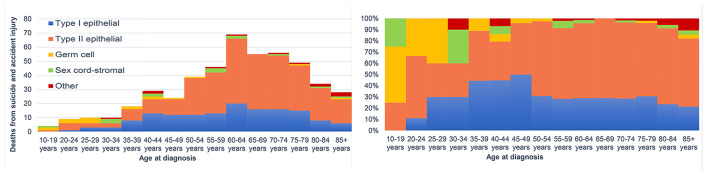
The number of suicides and accidental death among patients with ovarian cancer by age at diagnosis.

There were 27 suicides and 77 accidental deaths among patients with ovarian cancer who were younger than 50 years old. These younger cancer patients had almost the same risk of suicide as the general population with the same distribution of age, sex, and race [SMR = 1.25; 95% CI (0.86–1.83)]. But a significantly higher incidence of suicide and accidental death in patients over 50 was observed. Among cancer patients diagnosed between 50 and 59 years old, there were 36 suicides of 32,298 patients, accounting for 33.6% of all suicides among patients with ovarian cancer (Table 2). While for patients whose age at diagnosis was more than 50 years old, the suicide risk gradually decreased with age but increased after the age of 80. Figure 3 showed the number and proportion of suicides and accidental deaths in patients with ovarian cancer of different histological subtypes at different ages.

**Table 2:** The risk of suicide and accidental death among patients with ovarian cancer by age at diagnosis.

**Variable**	**Suicides**					**Accidental deaths**				
	**Patients with ovarian cancer**			**General population**		**Patients with ovarian cancer**			**General population**	
	**Observed deaths,** **No. (%)**	**Mortality rate^a^**	**SMR (95%CI)**	**Expected deaths**	**Mortality rate^a^**	**Observed deaths,** **No. (%)**	**Mortality rate^a^**	**SMR (95%CI)**	**Expected deaths**	**Mortality rate^a^**
**Age**										
0–20	2 (2)	8.20	3.45 (0.86–13.81)	0.58	2.37	2 (1)	8.20	0.54 (0.13–2.15)	3.71	15.21
20–29	5 (5)	11.96	2.21 (0.92–5.32)	2.26	5.40	14 (4)	33.50	1.79 (1.06–3.02)	7.82	18.71
30–39	7 (7)	9.52	1.38 (0.66–2.89)	5.07	6.90	21 (6)	28.57	1.61 (1.05–2.47)	13.06	17.77
40–49	13 (12)	8.59	0.95 (0.55–1.64)	13.61	9.00	40 (11)	26.43	1.25 (0.92–1.71)	31.97	21.12
50–59	36 (34)	18.22	1.98 (1.43–2.74)	18.19	9.21	49 (13)	24.80	1.08 (0.82–1.43)	45.41	22.99
60–69	26 (24)	16.55	2.37 (1.62–3.49)	10.95	6.97	98 (26)	62.36	2.38 (1.95–2.90)	41.16	26.19
70–79	13 (12)	13.99	2.47 (1.43–4.25)	5.26	5.66	92 (25)	99.00	1.91 (1.56–2.35)	48.06	51.71
80+	5 (5)	16.57	3.40 (1.41–8.16)	1.47	4.88	57 (15)	188.85	1.13 (0.87–1.46)	50.60	167.63

*SMR, standardized mortality ratio; CI, confidence intervals*.

a *Per 100,000 person-years. The mortality rate of the general population was calculated as the ratios of expected deaths from suicide or accidental injury to person-years of relevant strata in the cancer-free group*.

Among all patients with ovarian cancer, white patients had the highest risk of suicide [SMR = 1.82; 95% CI (1.50–2.23)] and black patients had the highest risk of accidental death [SMR = 1.87; 95% CI (1.27–2.75)]. The predominant patients who committed suicide were those with high education level (42, 39%), high income (43, 40%), and metropolitan residents (97, 91%), with an SMR of 2.12 [95% CI (1.57–2.87)], 2.05 [95% CI (1.52–2.77)], and 1.93 [95% CI (1.58–2.35)], respectively.

### Tumor-Related Characteristics Associated With Higher Risk of Suicide and Accidental Death

Patients with high-grade ovarian cancer had a higher risk of suicide than those with low-grade ovarian cancer [17.88 /100,000 person-years; SMR = 2.37; 95% CI (1.77–3.17) vs. 7.23/100,000 person-years; SMR = 0.94; 95% CI (0.59–1.49)], while there was no difference in the risk of accidental death among the two groups [44.10/100,000 person-years; SMR = 1.32; 95% CI (1.10–1.59) and 41.78 /100,000 person-years; SMR = 1.48; 95% CI (1.22–1.80)]. Patients with regional cancer and those with distant cancer were more likely to die from suicide [15.19 /100,000 person-years; SMR = 2.02; 95% CI (1.24–3.30) and 16.02/100,000 person-years; SMR = 2.15; 95% CI (1.63–2.85)], while patients with localized ovarian cancer [9.82/100,000 person-years; SMR = 1.31; 95% CI (0.92–1.85)] had the same risk of suicide as their matched general population. Moreover, patients with distant ovarian cancer had the highest risk of accidental death [54.91 /100,000 person-years; SMR = 1.59; 95% CI (1.37–1.85)], and patients with localized ovarian cancer and those with regional ovarian cancer had the same risk of accidental death [41.11 /100,000 person-years; SMR = 1.50; 95% CI (1.27–1.78); 44.61/100,000 person-years; 95% CI (1.05–1.86)].

### Tumor Treatment Associated With Higher Suicidal and Accidental Death Risk

The suicide rate of patients who did not undergo surgery for ovarian cancer [SMR = 6.75; 95% CI (4.07–11.20)] was much greater than that among those who underwent surgery [SMR = 1.57; 95% CI (1.27–1.94)]. Among women who received surgical treatment, those who underwent pelvic exenteration had the least number of cases but the highest risk of suicide [29.67/100,000 person-years; SMR = 3.88; 95% CI (1.25–12.03)]. Patients who underwent oophorectomy with hysterectomy had a higher risk of suicide as compared to those who underwent oophorectomy without hysterectomy [11.24 /100,000 person-years; SMR = 1.42; 95% CI (1.01–2.00) vs. 6.82/100,000 person-years; SMR = 1.09; 95% CI (0.52–2.28)]. In addition, patients who did not undergo surgery for ovarian cancer and those who underwent oophorectomy with hysterectomy had a higher risk of accidental death compared with their matched general population [149.88/100,000 person-years; SMR = 2.32; 95% CI (1.77–3.04) and 40.20/100,000 person-years; SMR = 1.37; 95% CI (1.14–1.64)].

### Suicidal and Accidental Death Risk in Different Histological Subtypes of Ovarian Cancer

**Figure 4:**
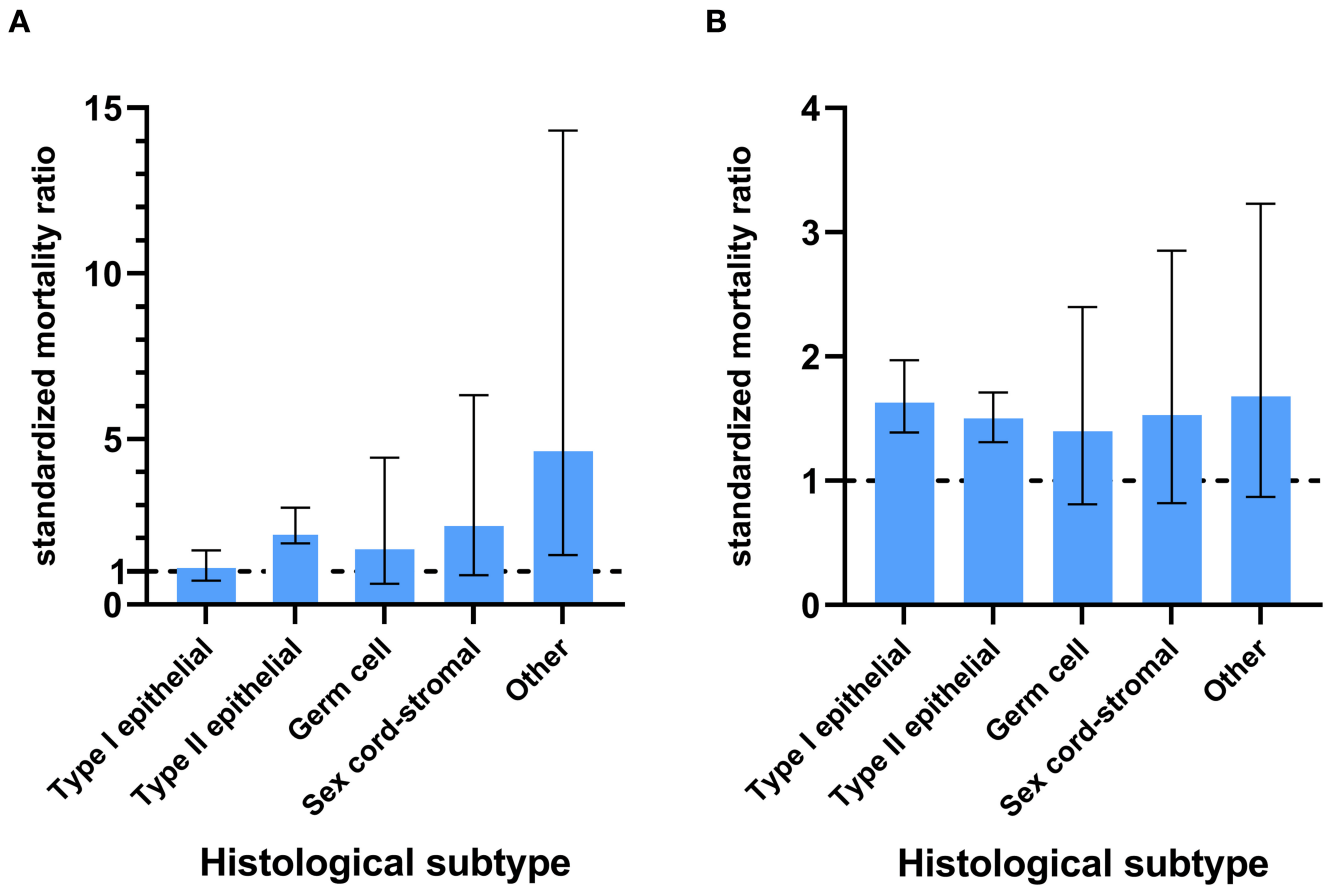
The SMRs with 95% CIs for patients with different subtypes of ovarian cancer. The lower limit of the CI exceeding 1 means that the patients had a higher risk of suicide **(A)** or accidental death **(B)** compared with the general population.

Only patients with Type II epithelial ovarian cancer had an increased risk of suicide, and those with Type I and Type II epithelial ovarian cancer were at a higher risk of accidental death compared with the general population (Figure 4). Among 102,418 patients with type II ovarian cancer, there were 72 suicides and 214 accidental deaths, accounting for 67 and 57% of all suicides and accidental deaths among ovarian cancer patients, respectively (Table 3). These patients had the highest risk of suicide and accidental deaths among all patients with ovarian cancer [17.49 /100,000 person-years; SMR = 2.31; 95% CI (1.83–2.91) and 51.98/100,000 person-years; SMR = 1.49; 95% CI (1.30–1.70)], followed by those with sex cord ovarian cancer for suicide [16.55 /100,000 person-years; SMR = 2.37; 95% CI (0.89–6.32)] and those with type I ovarian cancer for accidental death [46.68/100,000 person-years; SMR = 1.65; 95% CI (1.39–1.97)]. Patients with ovarian cancer of sex cord, type I, and germ cell had an almost equal risk of suicide [16.55/100,000 person-years; SMR = 2.37; 95% CI (0.89–6.32), 8.82 /100,000 person-years; SMR = 1.12; 95% CI (0.75–1.66), and 7.84/100,000 person-years; SMR = 1.67; 95% CI (0.63–4.44)] and patients with ovarian cancer of sex cord and germ cell had an almost equal risk of accidental death compared with the general population [41.39/100,000 person-years; SMR = 1.53; 95% CI (0.82–2.85) and 25.48/100,000 person-years; SMR = 1.40; 95% CI (0.81–2.40)]. For type II ovarian cancer patients, those with undifferentiated ovarian cancer had the highest risk of suicide and accidental death (23.94/100,000 person-years; SMR = 3.26; 95% CI (2.18–4.86) and 77.80/100,000 person-years; SMR = 1.94; 95% CI (1.56–2.43)], followed by those with high-grade serous [15.24/100,000 person-years; SMR = 1.99; 95% CI (1.48–2.69) and 43.23/100,000 person-years; SMR = 1.30; 95% CI (1.09–1.56)] and mixed epithelial-stromal carcinoma [17.11/100,000 person-years; SMR = 2.25; 95% CI (0.94–5.41) and 47.90/100,000 person-years; SMR = 1.39; 95% CI (0.82–2.35)].

**Table 3:** The risk of suicide and accidental death among patients with ovarian cancer by histological subgroup.

**Histological group**	**No. of patients, *n* (%)**	**Person-years**	**Suicides**			**Accidental deaths**		
			**No. of deaths, *n* (%)**	**Mortality rate**	**SMR (95%CI)**	**No. of deaths, *n* (%)**	**Mortality rate**	**SMR (95%CI)**
**Type I epithelial**	34,289 (23)	272,065.13	24 (22)	8.82	1.12 (0.75–1.66)	127 (34)	46.68	1.65 (1.39–1.97)
Low-grade serous carcinoma	980 (1)	4,423.38	1 (1)	22.61	2.94 (0.41–20.89)	4 (1)	90.43	2.82 (1.06–7.52)
Clear cell carcinoma	6,598 (4)	45,358.08	2 (2)	4.41	0.55 (0.14–2.20)	15 (4)	33.07	1.22 (0.73–2.02)
Endometrioid carcinoma	14,699 (10)	124,382.46	10 (9)	8.04	0.99 (0.53–1.84)	52 (14)	41.81	1.47 (1.12–1.93)
Mucinous carcinoma	10,885 (7)	91,424.29	10 (9)	10.94	1.44 (0.78–2.68)	50 (13)	54.69	1.97 (1.50–2.61)
Squamous carcinoma	489 (0)	2,336.75	0 (0)	0.00	0.00 (–)	2 (1)	85.59	3.03 (0.76–12.11)
Transitional cell or Brenner carcinoma	638 (0)	4,140.17	1 (1)	24.15	3.31 (0.47–23.50)	4 (1)	96.61	2.40 (0.90–6.39)
**Type II epithelial**	102,418 (69)	411,700.75	72 (67)	17.49	2.31 (1.83–2.91)	214 (57)	51.98	1.49 (1.30–1.70)
High-grade serous carcinoma	58,242 (39)	282,216.67	43 (40)	15.24	1.99 (1.48–2.69)	122 (33)	43.23	1.30 (1.09–1.56)
Mixed epithelial-stromal carcinoma	7,464 (5)	29,225.54	5 (5)	17.11	2.25 (0.94–5.41)	14 (4)	47.90	1.39 (0.82–2.35)
Undifferentiated or other epithelial	36,712 (25)	100,258.54	24 (22)	23.94	3.26 (2.18–4.86)	78 (21)	77.80	1.94 (1.56 −2.43)
Germ cell	4,436 (3)	51,028.00	4 (4)	7.84	1.67 (0.63–4.44)	13 (3)	25.48	1.40 (0.81–2.40)
Sex cord-stromal	2,620 (2)	24,162.46	4 (4)	16.55	2.37 (0.89–6.32)	10 (3)	41.39	1.53 (0.82–2.85)
**Others**	5,441 (4)	9,883.83	3 (3)	30.35	4.62 (1.49–14.32)	9 (2)	91.06	1.68 (0.87–3.23)
Non-specific	5,075 (3)	8,386.29	2 (2)	23.85	3.66 (0.91–14.62)	9 (2)	107.32	1.83 (0.95–3.51)
Other specific non-epithelial	366 (0)	1,497.54	1 (1)	66.78	9.75 (1.37–69.20)	0 (0)	0.00	0.00 (–)

*SMR, standardized mortality ratio; CI, confidence intervals*.

### Internal Comparisons: Predictors of Suicide and Accidental Death Based on Fine-Gray Model

**Figure 5:**
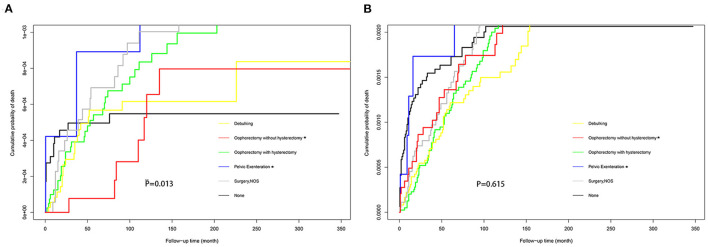
Cumulative probability of suicide **(A)** and accidental death **(B)** in women with ovarian cancer who received different types of surgery.

The Fine-Gray model revealed that age was found to be a protective factor for suicide [HR = 0.98; 95% CI (0.97–0.99); *P* < 0.001] but an independent risk factor for accidental death [HR = 1.02; 95% CI (1.01–1.03); *P* < 0.001] among patients with ovarian cancer (Table 4). Patients with high-grade ovarian cancer had a higher risk of suicide rather than accidental death than those with low-grade ovarian cancer. Besides, patients with localized [HR = 1.85; 95% CI (1.11–3.08); *P* = 0.019 and HR = 2.08; 95% CI (1.56–2.78); *P* < 0.001] or regional ovarian cancer [HR = 1.97; 95% CI (1.12–3.47); *P* = 0.019 and HR = 1.65; 95% CI (1.19–2.19); *P* = 0.003] had relatively higher risk of suicide and accidental death compared with those with distant ovarian cancer. The risk of suicide among patients who underwent pelvic exenteration [HR = 4.14; 95% CI (1.07–15.98); *P* = 0.039] for ovarian cancer was approximately five times that of patients who underwent oophorectomy without hysterectomy, while there was no statistical difference in the risk of accidental death in the two groups [HR = 1.03; 95% CI (0.40–2.62); *P* = 0.960]. Figure 5 showed the cumulative incidence of suicide and accidental death among patients who underwent different types of surgery throughout follow-up. Moreover, patients with localized or regional ovarian cancer had a significantly high risk of suicide and accidental death. The high-grade cancer population had a higher rate of suicide risk while the risk of accidental death was the same as that of the low-grade cancer population.

**Table 4:** Fine and Gray model for the risk of suicide and accidental death among patients with ovarian cancer.

**Variable**	**Suicides**		**Accidental death**	
	**HR (95%CI)**	***P***	**HR (95%CI)**	***P***
Age at diagnosis	0.98 (0.97–0.99)	<0.001	1.02 (1.01–1.03)	<0.001
**Race**				
Black	Ref.		Ref.	
White	4.62 (1.12–19.00)	0.034	0.95 (0.64–1.42)	0.810
Other	3.16 (0.64–15.58)	0.160	0.58 (0.30–1.11)	0.098
Year of diagnosis	0.98 (0.96–1.00)	0.069	0.98 (0.97–0.99)	<0.001
**Education**				
High	Ref.		Ref.	
Medium	0.76 (0.41–1.41)	0.380	1.10 (0.78–1.54)	0.590
Low	0.84 (0.39–1.84)	0.670	0.91 (0.61–1.37)	0.660
**Income**				
High	Ref.		Ref.	
Medium	0.90 (0.46–1.74)	0.750	1.06 (0.74–1.51)	0.760
Low	1.14 (0.56–2.32)	0.720	1.11 (0.76–1.63)	0.580
**Residence**				
Metropolitan	Ref.		Ref.	
Non-metropolitan	0.46 (0.19–1.14)	0.093	1.06 (0.75–1.50)	0.750
Unknown	3.21 (0.98–10.51)	0.054	0.95 (0.37–2.40)	0.910
**Grade**				
Low	Ref.		Ref.	
High	2.08 (1.17–3.70)	0.013	0.80 (0.60–1.06)	0.120
Unknown	1.46 (0.80–2.68)	0.220	0.92 (0.69–1.22)	0.550
**Stage**				
Localized	1.85 (1.11–3.08)	0.019	2.08 (1.56–2.78)	<0.001
Regional	1.97 (1.12–3.47)	0.019	1.65 (1.19–2.29)	0.003
Distant	Ref.		Ref.	
Unknown	2.09 (0.99–4.45)	0.055	1.29 (0.82–2.02)	0.270
**Surgery**				
Oophorectomy without hysterectomy	Ref.		Ref.	
Debulking	2.12 (0.86–5.25)	0.100	0.82 (0.54–1.27)	0.380
Oophorectomy with hysterectomy	2.05 (0.90–4.66)	0.088	1.07 (0.74–1.53)	0.730
Pelvic Exenteration	4.14 (1.07–15.98)	0.039	1.03 (0.40–2.62)	0.960
Surgery, NOS	1.68 (0.66–4.27)	0.270	0.97 (0.62–1.51)	0.890
None	2.40 (0.90–6.39)	0.081	0.72 (0.44–1.17)	0.190
Unknown	2.32 (0.68–7.96)	0.180	0.67 (0.36–1.25)	0.210
**Chemotherapy**				
None/unknown	Ref.		Ref.	
Yes	0.74 (0.48–1.14)	0.170	0.93 (0.74–1.16)	0.520
**Radiation**				
None/unknown	Ref.		Ref.	
Yes	1.22 (0.58–2.58)	0.600	0.86 (0.54–1.38)	0.530

*HR, hazard ratio; CI, confidence intervals*.

### Suicidal and Accidental Death Risk Over Time After Diagnosis

The relative increase in suicide risk among ovarian cancer patients was the highest within the first year after initial diagnosis and subsided with longer follow-up time (Table 5). Ten years after the cancer diagnosis, the suicide risk of cancer patients was equal to that of the general population. The SMR for accidental death declined within the first 5 years of cancer diagnosis and then elevated after 5 years (Table 6). The highest relative risks of suicide and accidental death persisted over time in patients with type II ovarian cancer.

**Table 5:** The risk of suicide among patients with ovarian cancer by histological group and time since diagnosis.

**Histological group**	**Time since diagnosis**			
	**0–1 year**	**1–5 years**	**5–10 years**	**10+ years**
**All**				
No. of suicides	26	48	20	13
Person-years accrued	117,513.25	276,228.4167	171,771.1667	211,894.5
SMR	3.09	2.35	1.54	0.80
95%CI	2.10–4.54	1.77–3.12	1.00–2.39	0.46–1.38
**Type I epithelial**				
No. of suicides	1	11	7	5
Person-years accrued	29,950.21	85,202.08	67,796.92	91,390.25
SMR	0.44	1.66	1.31	0.67
95%CI	0.06–3.11	0.92–3.00	0.62–2.74	0.28–1.62
**Type II epithelial**				
No. of suicides	23	35	10	4
Person-years accrued	78,655.08	166,094.58	83,175.42	89,416.83
SMR	4.09	2.83	1.56	0.56
95%CI	2.72–6.15	2.03–3.94	0.84–2.89	0.21–1.49
**Germ cell**				
No. of suicides	0	0	0	4
Person-years accrued	4,090.75	13,506.58	12,457.75	21,296.92
SMR	0	0	0	4.10
95%CI	–	–	–	1.54–10.91
**Sex cord-stromal**				
No. of suicides	1	0	3	0
Person-years accrued	2,417.88	7,592.83	6,307.83	8,033.50
SMR	6.23	0	6.84	0
95%CI	0.88–44.20	–	2.21–21.22	–
**Others**				
No. of suicides	1	2	0	0
Person-years accrued	2,399.33	3,832.33	2,033.25	1,757.00
SMR	6.87	7.96	0	0
95%CI	0.97–48.75	1.99–31.84	–	–

*SMR, standardized mortality ratio; CI, confidence intervals*.

**Table 6:** The risk of accidental death among patients with ovarian cancer by histological group and time since diagnosis.

	**Time since diagnosis**			
**Histological group**	**0–1 year**	**1–5 years**	**5–10 years**	**10+ years**
**All**				
No. of accidental deaths	73	110	67	123
Person-years accrued	117,513.25	276,228.4167	171,771.1667	211,894.5
SMR	1.48	1.15	1.34	2.46
95%CI	1.18–1.86	0.95–1.38	1.06–1.70	2.06–2.93
**Type I epithelial**				
No. of accidental deaths	12	35	23	57
Person-years accrued	29,950.21	85,202.08	67,796.92	91,390.25
SMR	1.19	1.32	1.20	2.62
95%CI	0.68–2.10	0.95–1.84	0.80–1.81	2.02–3.40
**Type II epithelial**				
No. of accidental deaths	54	65	38	57
Person-years accrued	78,655.08	166,094.58	83,175.42	89,416.83
SMR	1.52	1.04	1.45	2.55
95%CI	1.17–1.99	0.82–1.33	1.06–2.00	1.97–3.31
**Germ cell**
No. of accidental deaths	2	4	2	5
Person-years accrued	4,090.75	13,506.58	12,457.75	21,296.92
SMR	2.51	1.56	0.88	1.34
95%CI	0.63–10.04	0.59–4.16	0.22–3.51	0.56–3.21
**Sex cord-stromal**				
No. of accidental deaths	1	2	3	4
Person-years accrued	2,417.88	7,592.83	6,307.83	8,033.50
SMR	1.31	0.88	1.75	2.18
95%CI	0.19–9.33	0.22–3.52	0.56–5.42	0.82–5.80
**Others**				
No. of accidental deaths	4	4	1	0
Person-years accrued	2,399.33	3,832.33	2,033.25	1,757.00
SMR	1.85	1.82	1.44	0
95%CI	0.70–4.94	0.68–4.86	0.20–10.19	–

*SMR, standardized mortality ratio; CI, confidence intervals*.

## Discussion

### Main Findings

Using data from the SEER database, we first found that a relative risk of 1.9 for suicide and 1.5 for accidental death among women with ovarian cancers compared with the general US women population. Of note, we found that patients with Type II epithelial ovarian cancer faced an increased risk of both suicide and accidental deaths in comparison with the general population, and the patients with Type I epithelial ovarian cancer also had a greater risk of accidental deaths. Given that suicide is potentially preventable, this analysis is important as it identifies the high-risk groups of death from suicide and accidental injury among ovarian cancer patients, providing a preliminary heuristic framework for clinicians to develop suicide risk-identification and prevention strategies to reduce the risk of suicide among women with ovarian cancer.

### Strengths and Limitations

Despite many important findings of an association between ovarian cancer and the risk of suicide and accidental death, there are some limitations in our study. First, the SEER database does not contain detailed information regarding chemoradiotherapy such as cycles and dosage, which renders it difficult to evaluate the impact of chemoradiotherapy on the risk of suicide and accidental death. Second, data on psychiatric status, quality of life, and social support are also unavailable in the SEER database. Third, data on a history of psychiatric disorders, complications of cancer and treatment, the quality of life, and social support are also unavailable in the SEER database, therefore, we cannot assess the direct association between these factors and the risk of suicide and accidental death among patients with ovarian cancer. Nevertheless, this study is the first large population-based study on the risk of suicide and accidental death in patients with ovarian cancer. Hence, the results of the study are reliable and applicable to the rest of the population.

### Interpretation

Some of our research results are very meaningful. Our study showed that patients with type II epithelial ovarian cancer faced a greater risk of suicidal and accidental mortality which may be attributed to the advanced cancer stage and poor prognosis compared with type I epithelial, germ cell, and sex cord-stromal ovarian cancer (16, 17). A Danish study containing 2660 cases reported that 78.1% of patients with type II epithelial ovarian cancer were diagnosed in late cancer stages, with a median follow-up time of 24 months; as a comparison, only 32.1% of patients with type I epithelial ovarian cancer diagnosed in late cancer stages, with a median follow-up time of 36 months (18). Using the SEER database, Edward et al. found that the disease-specific survival of type II is being 64–34% of type I over the 200-month range (16). In the context of the poor prognosis, patients with type II epithelial sarcoma would suffer more psychological concerns, including anxiety and depression. They could feel isolated and hopeless due to the lack of survivor groups. All these may lead to intense suicidal ideation among women with type II epithelial ovarian cancer.

We also found that the risk of suicide was the highest within the first year after initial diagnosis among patients with ovarian cancer. This is a novel finding of the present study, and our results stand corroborated by other cancer studies which showed an elevated risk of suicide within the first year of diagnosis compared with cancer-free controls. Mahdi et al. reported the highest suicide rates within the first year following diagnosis among women with gynecologic cancer, with an SMR of 2.8 (8). Further, prior research has shown that significantly higher risks of suicide were observed among patients diagnosed with breast cancer (19), brain cancer (20), male genital-system (21), and skin malignant melanoma (22) within the first year of diagnosis. We thought it reasonable as the change from being a healthy individual to being a cancer patient who had a poor prognosis created a new perception of identity. During this process of shift, patients with ovarian cancer often suffer from severe psychological distress and problems in social life (23). This novel finding underlines the concept that healthcare providers should better understand the social psychology and identity changes of newly diagnosed patients with ovarian cancer to better guide ovarian cancer survivors during follow-up. Many methods could be utilized to evaluate the level of mental health and suicidal ideation among cancer patients, such as item 9 of the Patient Health Questionnaire depression module, Center for Epidemiologic Studies Depression Scale (CES-D), the Impact of Event Scale (IES), and the Profile of Mood States short-form (POMS-SF) (24).

Within the ovarian cancer cohort, we observed that those who underwent pelvic exenteration were at the highest risk of suicide. Pelvic exenteration is a radical surgical procedure that removes the visceral pelvic organs including the uterus, tubes, ovaries, parametrium, vagina, urinary bladder, urethra, and rectum, with or without the perineum in an en-bloc fashion (25–27). It is considered the last curative opportunity for malignant gynecological tumors (28). However, it is related to various complications, high costs of substantial treatment, and high mortality. A survey conducted in New Zealand reported 106 complications, such as intra-abdominal collection (43.7%) and wound infection (14.1%), out of a total of 646 consecutive patients who required extended surgery for local advanced pelvic malignancies (29). Moreover, severe gastrointestinal and urinary tract symptoms such as dyspareunia, as well as decreased sexual functions and sexual desire, have also been observed among ovarian cancer patients, especially for those who underwent pelvic exenteration (30, 31). These adverse effects and accompanying symptoms had an obvious negative impact on global health status, body image, self-identity, social functioning, emotional response, and quality of life among these survivors (32). Therefore, these survivors who underwent pelvic exenteration have an increased risk of suicide and need close monitoring, and unnecessary operations should be avoided to reduce the risk of suicide in patients with ovarian cancer (33, 34).

Ovarian cancer occurs more in patients older than 50 years, similarly, and we found that patients diagnosed over 50 were at higher risk of suicide, especially between the ages of 50–59. Our results stand corroborated by Stephanie's study which found that female patients with cancer whose age at diagnosis between 55 and 59 had the highest suicide risk among all female cancer survivors (5). Furthermore, it was well-recognized that older age was associated with a higher risk of suicide among patients with prostate cancer, lung cancer (35), colorectal cancer, and bladder cancer (36). The working hypotheses for our finding may be that ovarian cancer patients with older age may suffer more severe emotional and psychological distress. Payne reported that some symptoms, such as the anxiety and depression of recurrent disease and death, as well as sleep disorders, may persist longer in elder patients (37). An Australian survey using the Insomnia Severity Index (ISI) to assess the degree of insomnia also confirmed that ovarian cancer patients aged 50–59 present had clinically higher levels of insomnia (38), which has been proven to be correlated with suicidal thoughts and attempts (39). Moreover, older patients could get less information about cancer and psychological support than younger patients from the internet (40, 41), therefore, they lack the confidence to defeat cancer (42), leading to relatively higher suicidal ideation (43). Given the great difference in suicide risk between young and old survivors with ovarian cancer, it is necessary to regard old patients as a distinctive group that warrants special attention when considering the suicide risk of cancer patients.

In conclusion, our study demonstrated that patients with ovarian cancer, especially type II ovarian cancer, had an increased relative risk of suicide and accidental death to that of the cancer-free population. Further, it is worth noting that pelvic exenteration for ovarian cancer would increase the risk of suicide, but not increase the risk of accidental death. These meaningful findings suggest a need in clinical work to detect suicidal ideation as early as possible especially for high-risk subgroups (44, 45). We can develop an accurate predictive model to predict the risk of suicide and accidental death in patients and to pay more attention to the high-risk group, and appropriate psychosocial interventions and treatment are also essential, as clinicians could provide psychological counseling to cancer patients to reduce their anxiety, depression and loneliness, and strengthen their confidence to face cancer and help them return to community life, such as psychosocial and emotional support through interpersonal interactions during survivorship care, which potentially reduce the risk of suicide in cancer survivors. In this way, a better balance can be achieved between achieving a better overall prognosis and reducing the risk of suicide and accidental death (46, 47). Future studies should explore the direct relationship between psychosocial factors and the risk of suicide and accidental death among ovarian cancer patients, which may discover new interventions that can improve the survival of patients diagnosed with ovarian cancer.

## Data Availability Statement

The original contributions presented in the study are included in the article/supplementary material, further inquiries can be directed to the corresponding author/s.

## Author Contributions

SW and MW designed this study. YC, KY, and JX drafted the manuscript. JZ, SZ, and JD prepared all the figures and tables. All authors contributed to the article and approved the submitted version.

## Funding

This work was supported by grants from the National Natural Science Foundation of China (Nos. 81873824 and 82001514) and the Fundamental Research Funds for the Central Universities (HUST: 2021yjsCXCY087).

## Conflict of Interest

The authors declare that the research was conducted in the absence of any commercial or financial relationships that could be construed as a potential conflict of interest.

## Publisher's Note

All claims expressed in this article are solely those of the authors and do not necessarily represent those of their affiliated organizations, or those of the publisher, the editors and the reviewers. Any product that may be evaluated in this article, or claim that may be made by its manufacturer, is not guaranteed or endorsed by the publisher.

## References

[1] Centers for Disease Control and Prevention Preventing suicide. National Center for Injury Prevention and Control, Division of Violence Prevention (2018). Available from: https://www.cdc.gov/violenceprevention/suicide/fastfact.html

[2] Pritchard C Hean S. Suicide and undetermined deaths among youths and young adults in Latin America: comparison with the 10 major developed countries–a source of hidden suicides? Crisis. (2008) 29:145–53. 10.1027/0227-5910.29.3.145 18714911

[3] Rockett IR Hobbs G De Leo D Stack S Frost JL Ducatman AM. Suicide and unintentional poisoning mortality trends in the United States, 1987-2006: two unrelated phenomena? BMC Public Health. (2010) 10:705. 10.1186/1471-2458-10-705 21083903 PMC3091585

[4] Bergen H Hawton K Kapur N Cooper J Steeg S Ness J. Shared characteristics of suicides and other unnatural deaths following non-fatal self-harm? A multicentre study of risk factors. Psychol Med. (2012) 42:727–41. 10.1017/S0033291711001747 21910932

[5] Misono S Weiss NS Fann JR Redman M Yueh B. Incidence of suicide in persons with cancer. J Clin Oncol. (2008) 26:4731–8. 10.1200/JCO.2007.13.8941 18695257 PMC2653137

[6] Yang K Zheng Y Peng J Chen J Feng H Yu K. Incidence of death from unintentional injury among patients with cancer in the United States. JAMA Netw Open. (2020) 3:e1921647. 10.1001/jamanetworkopen.2019.21647 32083692 PMC7043194

[7] Ward KK Roncancio AM Plaxe SC. Women with gynecologic malignancies have a greater incidence of suicide than women with other cancer types. Suicide Life Threat Behav. (2013) 43:109–15. 10.1111/sltb.12002 23278597 PMC3955113

[8] Mahdi H Swensen RE Munkarah AR Chiang S Luhrs K Lockhart D. Suicide in women with gynecologic cancer. Gynecol Oncol. (2011) 122:344–9. 10.1016/j.ygyno.2011.04.015 21561646

[9] Doubeni CA Doubeni AR Myers AE. Diagnosis and management of ovarian cancer. Am Fam Physician. (2016) 93:937–44. 27281838

[10] Nakao S Minaguchi T Itagaki H Hosokawa Y Shikama A Tasaka N. Pretreatment thrombocytosis as an independent predictive factor for chemoresistance and poor survival in epithelial ovarian cancer. J Ovarian Res. (2020) 13:55. 10.1186/s13048-020-00651-6 32375852 PMC7201937

[11] Dalela D Krishna N Okwara J Preston MA Abdollah F Choueiri TK. Suicide and accidental deaths among patients with non-metastatic prostate cancer. BJU Int. (2016) 118:286–97. 10.1111/bju.13257 26305451

[12] National Cancer Institute. Surveillance Epidemiology, and End Results Program. Available online at: http://www.seer.cancer.gov (accessed April 1, 2020).

[13] Peres LC Cushing-Haugen KL Kobel M Harris HR Berchuck A Rossing MA. Invasive epithelial ovarian cancer survival by histotype and disease stage. J Natl Cancer Inst. (2019) 111:60–8. 10.1093/jnci/djy071 29718305 PMC6335112

[14] Matz M Coleman MP Carreira H Salmeron D Chirlaque MD Allemani C. Worldwide comparison of ovarian cancer survival: Histological group and stage at diagnosis (CONCORD-2). Gynecol Oncol. (2017) 144:396–404. 10.1016/j.ygyno.2016.11.019 27919574 PMC6195190

[15] Koepsell TD Weiss NS. Epidemiologic Methods: Studying the Occurrence of Illness. New York, NY: Oxford University Press (2003).

[16] Pavlik EJ Smith C Dennis TS Harvey E Huang B Chen Q. Disease-specific survival of type I and type II epithelial ovarian cancers-stage challenges categorical assignments of indolence & aggressiveness. Diagnostics. (2020) 10:56–67. 10.3390/diagnostics10020056 31973035 PMC7168156

[17] Matz M Coleman MP Sant M Chirlaque MD Visser O Gore M. The histology of ovarian cancer: worldwide distribution and implications for international survival comparisons (CONCORD-2). Gynecol Oncol. (2017) 144:405–13. 10.1016/j.ygyno.2016.10.019 27931752 PMC6195192

[18] Prahm KP Karlsen MA Hogdall E Scheller NM Lundvall L Nedergaard L. The prognostic value of dividing epithelial ovarian cancer into type I and type II tumors based on pathologic characteristics. Gynecol Oncol. (2015) 136:205–11. 10.1016/j.ygyno.2014.12.029 25546113

[19] Gaitanidis A Alevizakos M Pitiakoudis M Wiggins D. Trends in incidence and associated risk factors of suicide mortality among breast cancer patients. Psychooncology. (2018) 27:1450–6. 10.1002/pon.4570 29055289

[20] Saad AM Elmatboly AM Gad MM Al-Husseini MJ Jazieh KA Alzuabi MA. Association of brain cancer with risk of suicide. JAMA Netw Open. (2020) 3:e203862. 10.1001/jamanetworkopen.2020.3862 32356882 PMC7195621

[21] Yang J He G Chen S Pan Z Zhang J Li Y. Incidence and risk factors for suicide death in male patients with genital-system cancer in the United States. Eur J Surg Oncol. (2019) 45:1969–76. 10.1016/j.ejso.2019.03.022 30914288

[22] Yang J Chen S Li Y Wang B Xin X Xue X. Incidence rate and risk factors for suicide death in patients with skin malignant melanoma: a Surveillance, Epidemiology, and End Results analysis. Melanoma Res. (2020) 30:402–9. 10.1097/CMR.0000000000000559 30489483

[23] Webber K Carolus E Mileshkin L Sommeijer D McAlpine J Bladgen S. OVQUEST - Life after the diagnosis and treatment of ovarian cancer - An international survey of symptoms and concerns in ovarian cancer survivors. Gynecol Oncol. (2019) 155:126–34. 10.1016/j.ygyno.2019.08.009 31416612

[24] Davis LZ Cuneo M Thaker PH Goodheart MJ Bender D Lutgendorf SK. Changes in spiritual well-being and psychological outcomes in ovarian cancer survivors. Psychooncology. (2018) 27:477–83. 10.1002/pon.4485 28637083 PMC5740010

[25] de Gregorio N de Gregorio A Ebner F Friedl TWP Huober J Hefty R. Pelvic exenteration as ultimate ratio for gynecologic cancers: single-center analyses of 37 cases. Arch Gynecol Obstet. (2019) 300:161–8. 10.1007/s00404-019-05154-4 31011878

[26] Matsuo K Mandelbaum RS Adams CL Roman LD Wright JD. Performance and outcome of pelvic exenteration for gynecologic malignancies: a population-based study. Gynecol Oncol. (2019) 153:368–75. 10.1016/j.ygyno.2019.02.002 30792003 PMC7521603

[27] Chatterjee S Chen L Jones N Tergas AI Burke WM Hou JY. National trends in total pelvic exenteration for gynecologic malignancies. Am J Obstet Gynecol. (2016) 215:395–6. 10.1016/j.ajog.2016.06.031 27418447

[28] Romeo A Gonzalez MI Jaunarena J Zubieta ME Favre G Tejerizo JC. Pelvic exenteration for gynecologic malignancies: postoperative complications and oncologic outcomes. Actas Urol Esp. (2018) 42:121–5. 10.1016/j.acuroe.2017.12.007 28911880

[29] Peacock O Waters PS Kong JC Warrier SK Wakeman C Eglinton T. Complications after extended radical resections for locally advanced and recurrent pelvic malignancies: a 25-year experience. Ann Surg Oncol. (2020) 27:409–14. 10.1245/s10434-019-07816-8 31520213

[30] Korfage IJ Essink-Bot M-L Mols F van de Poll-Franse L Kruitwagen R van Ballegooijen M. Health-related quality of life in cervical cancer survivors: a population-based survey. Int J Radiat Oncol Biol Phys. (2009) 73:1501–9. 10.1016/j.ijrobp.2008.06.1905 18823716

[31] Cianci S Tarascio M Rosati A Caruso S Uccella S Cosentino F. Sexual function and quality of life of patients affected by ovarian cancer. Minerva Med. (2019) 110:320–9. 10.23736/S0026-4806.19.06080-4 31081305

[32] Dessole M Petrillo M Lucidi A Naldini A Rossi M De Iaco P. Quality of life in women after pelvic exenteration for gynecological malignancies: a multicentric study. Int J Gynecol Cancer. (2018) 28:267–73. 10.1097/IGC.0000000000000612 26807639

[33] Coleman RL Spirtos NM Enserro D Herzog TJ Sabbatini P Armstrong DK. Secondary surgical cytoreduction for recurrent ovarian cancer. N Engl J Med. (2019) 381:1929–39. 10.1056/NEJMoa1902626 31722153 PMC6941470

[34] Capozzi VA Rosati A Turco LC Sozzi G Riccò M Chiofalo B. Surgery vs. chemotherapy for ovarian cancer recurrence: what is the best treatment option. Gland Surg. (2020) 9:1112–7. 10.21037/gs-20-326 32953626 PMC7475368

[35] Urban D Rao A Bressel M Neiger D Solomon B Mileshkin L. Suicide in lung cancer: who is at risk? Chest. (2013) 144:1245–52. 10.1378/chest.12-2986 23681288

[36] Zaorsky NG Zhang Y Tuanquin L Bluethmann SM Park HS Chinchilli VM. Suicide among cancer patients. Nat Commun. (2019) 10:207. 10.1038/s41467-018-08170-1 30643135 PMC6331593

[37] Payne SA. A study of quality of life in cancer patients receiving palliative chemotherapy. Soc Sci Med. (1992) 35:1505–9. 10.1016/0277-9536(92)90053-S 1283035

[38] Price MA Zachariae R Butow PN. deFazio A Chauhan D Espie CA. Prevalence and predictors of insomnia in women with invasive ovarian cancer: anxiety a major factor. Eur J Cancer. (2009) 45:3262–70. 10.1016/j.ejca.2009.05.030 19540748

[39] Blank M Zhang J Lamers F Taylor AD Hickie IB Merikangas KR. Health correlates of insomnia symptoms and comorbid mental disorders in a nationally representative sample of US adolescents. Sleep. (2015) 38:197–204. 10.5665/sleep.4396 25325502 PMC4288600

[40] Bass SB Ruzek SB Gordon TF Fleisher L McKeown-Conn N Moore D. Relationship of Internet health information use with patient behavior and self-efficacy: experiences of newly diagnosed cancer patients who contact the National Cancer Institute's Cancer Information Service. J Health Commun. (2006) 11:219–36. 10.1080/10810730500526794 16537289

[41] Schiffman JD Csongradi E Suzuki LK. Internet use among adolescent and young adults (AYA) with cancer. Pediatr Blood Cancer. (2008) 51:410–5. 10.1002/pbc.21616 18506753

[42] Penn A Kuperberg A. Psychosocial Support In Adolescents And Young Adults With Cancer. Cancer J. (2018) 24:321–7. 10.1097/PPO.0000000000000339 30480577

[43] Zhou H Xian W Zhang Y Yang Y Fang W Liu J. Suicide among cancer patients: adolescents and young adult (AYA) versus all-age patients. Ann Transl Med. (2019) 7:658. 10.21037/atm.2019.10.51 31930059 PMC6944571

[44] Gupta KK Gupta VK Naumann RW. Ovarian cancer: screening and future directions. Int J Gynecol Cancer. (2019) 29:195–200. 10.1136/ijgc-2018-000016 30640704

[45] Rosati A Alletti SG Capozzi VA Mirandola M Vargiu V Fedele C. Role of ultrasound in the detection of recurrent ovarian cancer: a review of the literature. Gland Surg. (2020) 9:1092–101. 10.21037/gs-20-357 32953624 PMC7475345

[46] Chi DS Eisenhauer EL Zivanovic O Sonoda Y Abu-Rustum NR Levine DA. Improved progression-free and overall survival in advanced ovarian cancer as a result of a change in surgical paradigm. Gynecol Oncol. (2009) 114:26–31. 10.1016/j.ygyno.2009.03.018 19395008

[47] Marchetti C Rosati A De Felice F Boccia SM Vertechy L Pavone M. Optimizing the number of cycles of neoadjuvant chemotherapy in advanced epithelial ovarian carcinoma: a propensity-score matching analysis. Gynecol Oncol. (2021) 163:29–35. 10.1016/j.ygyno.2021.07.025 34312003

